# Ex vivo evaluation of an atherosclerotic human coronary artery via histology and high-resolution hard X-ray tomography

**DOI:** 10.1038/s41598-019-50711-1

**Published:** 2019-10-04

**Authors:** Marzia Buscema, Simone E. Hieber, Georg Schulz, Hans Deyhle, Alexander Hipp, Felix Beckmann, Johannes A. Lobrinus, Till Saxer, Bert Müller

**Affiliations:** 1 Biomaterials Science Center, Department of Biomedical Engineering, University of Basel, Allschwil, Switzerland; 2 Institute of Materials Research, Helmholtz-Zentrum Geesthacht, Geesthacht, Germany; 3 Neuropathology Unit, University Hospital of Geneva, Geneva, Switzerland; 4 Faculty of Medicine, University of Geneva, Geneva, Switzerland

**Keywords:** X-ray tomography, Biomedical engineering

## Abstract

Atherosclerotic arteries exhibit characteristic constrictions and substantial deviations from cylindrical shape. Therefore, determining the artery’s cross-section along the centerline is challenging, although high-resolution isotropic three-dimensional data are available. Herein, we apply high-resolution computed tomography in absorption and phase to a plaque-containing human artery *post*-*mortem*, through the course of the preparation stages for histology. We identify the impact of paraffin embedding and decalcification on the artery lumen. For automatic extraction of lumen’s cross-section along centerline we present a dedicated pipeline. Comparing fixated tissue before and after paraffin embedding gives rise to shape changes with lumen reduction to 50–80%. The histological slicing induces further deformations with respect to tomography. Data acquired after decalcification show debris unintentionally distributed within the vessel preventing the reliable automatic lumen segmentation. Comparing tomography of laboratory- and synchrotron-radiation-based X rays by means of joint histogram analysis leads us to conclude that advanced desktop tomography is capable of quantifying the artery’s lumen as an essential input for blood flow simulations. The results indicate that the most reliable lumen quantification is achieved by imaging the non-decalcified specimen fixed in formalin, using phase contrast modality and a dedicated processing pipeline. This study focusses on a methodology to quantitatively evaluate diseased artery segments *post*-*mortem* and provides unique structural parameters on the treatment-induced local shrinkage, which will be the basis of future studies on the flow in vessels affected by constrictions.

## Introduction

Blood vessels are commonly represented as a network of hollow tubes that transport blood through the human body. In a healthy situation, hemodynamics is characterized by a laminar flow and a wall shear stress in the order of 1 Pa^1^. On the contrary, the narrowing of blood vessels, for example as the result of atherosclerosis, gives rise to a wall shear stress increased by at least one order of magnitude^2^. Atherosclerosis, which belongs to serious disorders of the cardiovascular system, is a chronic disease caused by the build-up of white blood cells within the vascular wall leading to plaque formation^3^. Plaque rupture is responsible for acute myocardial infarction, also known as heart attack, which is among the main sources of mortality worldwide^4^.

Recent communications have proposed exploiting increased wall shear stress in constricted blood vessels for the targeted delivery of vasodilatory drugs^5,6^. For the clinical use, however, the threshold for drug release has to be determined. Consequently, the morphology of the vessel lumen in both healthy and diseased conditions has to be evaluated.

*In vivo* imaging techniques such as coronary computed tomography angiography and magnetic resonance imaging are used widely for the visualization and quantification of coronary artery occlusions. Although these techniques yield information under physiological conditions, they are limited in terms of the three-dimensional evaluation of blood vessel anatomy^7^, and they do not reach the micrometer precision required for meaningful flow simulations^8^. Therefore, researchers have applied expensive and time-consuming serial sectioning and the combination of two-dimensional micrographs^9^. This histological approach, however, relies on extended tissue preparation procedures, namely fixation, decalcification, embedding, and staining, which substantially modify the geometry of the vessel with respect to the *in vivo* situation.

It is well known that micro computed tomography (μCT) is a non-destructive imaging technique, which yields three-dimensional imaging data that can be, for example, used as a tool for selecting the planes for histological sectioning^10,11^. Several research teams have recently applied μCT for rendering diseased coronary arteries^12–15^.

Using the conventional absorption contrast, the plaque present in atherosclerotic vessels dominates X-ray absorption owing to its higher density with respect to the vessel wall^8^. At reduced photon energies, the calcium content of the plaque is highly X-ray absorbing, causing severe streak artefacts and compromising the visualization of the vessel wall. The cross-section in absorption scales with the fourth power of the atomic number, whereas the phase cross-section shows a linear behavior, as pointed out, for example, by A. Momose in Fig. 1 of ref.^16^. Therefore, hard X-ray phase contrast imaging is beneficial when the specimen contains high and low X-ray absorbing components simultaneously, as the case of an atherosclerotic vessel segment. Raising the absorption of the soft-tissue by appropriate stains to more closely match that of the plaque might ease the imaging of such a specimen in absorption contrast mode. Furthermore, the soft tissues, which consist of elements with a low atomic number, have a refractive index, where the real part, related to the phase shift, is three orders of magnitude larger than the imaginary part, related to the absorption^17^. This behavior explains the superior contrast obtained by tomography methods based on the phase shift of X rays^16^. This phase shift can be retrieved using crystal interferometry^18^, propagation-based approaches^19^, analyzer-based imaging^20^ and grating-based interferometry (XGI)^21^. Very recently, the relevant soft tissue components in an intact coronary artery segment have been made visible by means of the propagation-based hard X-ray phase tomography using an advanced small-spot laboratory X-ray source^22^. The authors observed microscopic lipid-rich plaques, adipose and foam cells, as well as the fibrous cap. This propagation-based approach provides true micrometer resolution and complements XGI, a method with restricted spatial resolution but superior density resolution^23^. Consequently, the present XGI study replenishes the report of Vågberg *et al*.^22^, because it sets more value on contrast than on true cellular resolution.

The tomography data were registered in order to determine local geometrical modifications. The lumen was segmented and the cross-section along the centerline derived. Centerlines are efficient representations of tubular objects such as blood vessels. In medical fields, they are important to quantify the morphology of vessels and trabecular bone. Whereas the centerlines can be efficiently determined in two dimensions, their extraction remains challenging in three dimension, particularly if the shape significantly deviates from a cylinder. Algorithms based on multiscale matched filter are available for the characterization of retinal vessels^24^. For the longitude of a bone, straight or curved in shape, a thinning-based approach was presented for obtaining its medial axis. The obtained medial axis captures the longitudinal centerline of the bone^25^.

Saha *et al*.^26^ reviewed the common skeletonization methods including center of maximal balls and distance transform approaches. A very recent approach^27^ shows the three-dimensional centerline extraction for discrete binary objects, in which the object is sliced into a series of two-dimensional images in the three orthogonal directions to determine the centerlines in two dimensions and to combine them. These techniques can result in misrepresenting branches for the complex morphology of the atherosclerotic artery. Machine learning approaches^28^ have been applied successfully to low-resolution images of vascular trees. But it remains unclear, whether the training of the proposed predictor variables can be accomplished on one dataset of limited size with complex outer surface. The centerline extraction is known to become difficult for branching over large structures. For a non-branching centerline of a centimeter-long blood vessel segment the task seems to be rather simple. Due to the complex shape of the vascular lumen within the constriction, however, the cross-section profile could be highly sensitive to the position and the bending of the centerline. Therefore, the unique definition of the centerline and its precise determination are crucial. Virtual histology on the basis of micro computed tomography in absorption and phase modes has been reported^11,29,30^, but a detailed study of a diseased artery in the sequence of the necessary workflow known from conventional histology, including decalcification, is missing. Herein, we report on μCT measurements and dedicated centerline evaluations of a diseased human artery segment subsequent to individual tissue preparation steps, i.e. formalin fixation, paraffin embedding, and decalcification.

## Results

### Artery imaging and lumen segmentation

**Figure 1:**
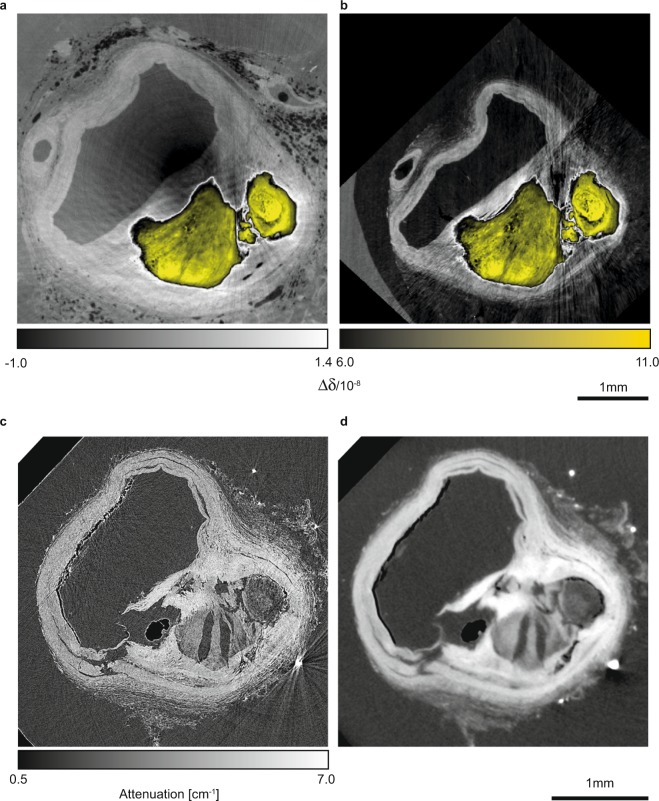
Selected slices from 3D-3D-registered CT-data before (**a**,**b**) and after decalcification (**c**,**d**) showing clearly the impact of water replacement. The phase contrast CT-slice from Dataset #1 (**a**) and the corresponding one from Dataset #2 (**b**) were acquired using a double grating interferometer. In the images, the Δ*δ* between the colorbars are represented by turquoise: the blue to yellow color represents the plaque, which allows for registration, as its size and shape hardly change, whereas the vessel walls given in light gray exhibit massive shrinkage. The absorption CT-slice from Dataset #3*A* (**c**) and the corresponding one from Dataset #3*B* (**d**) elucidate that lumen segmentation is a challenge and automatic procedures will probably fail due to vessel wall damage. Adapted from the manuscript of M.B.’s thesis^46^.

Table 1 details specimen preparation and the imaging parameters used. The image in Fig. 1a shows a CT-slice from Dataset #1, with its counterpart from Dataset #2 in Fig. 1b. The counterpart slice was identified by the three-dimensional affine registration of Datasets #1 and #2^31^. The similarity of the anatomical structures is elucidated best by the plaque, represented by a black to yellow color. Formalin fixation led to a dominant artefact, namely the dark area within the vessel wall, caused by the presence of gas bubbles within the artery lumen, as displayed in Fig. 1a.

**Table 1:** Specimen used and imaging parameters.

Label	Specimen preparation	Imaging parameters								
		Modality	X-ray source	*E* [keV]	Detector type (pixel used)	*l* [*μ*m]	*N*	*t* [s]	*T* [h]	*h*(*n*) [mm]
#1	Formalin fixation	XGI phase	P07, DESY	45	CMOS (5120 × 3840)	1.3	1800	0.2*	21.3	21.9 (10)
#2	Paraffin embedding	XGI phase	P07, DESY	45	CMOS (5120 × 3840)	1.3	1200	0.2*	15.6	13.9 (6)
#3A	Decalcified	absorption	P05, DESY	10	CCD (3056 × 3056)	2.4	1200	1.5^#^	9.3	10.3 (4)
#3B	Decalcified	absorption	conventional	≤15	CMOS (1944 × 1536)	5.2	1200	2.3^#^	6.9	16.6 (4)

*E*: photon energy; *l*: effective pixel length; *N*: number of projections; t*: exposure time per phase step image; *t*^#^: exposure time per projection image; *T*: total artery scan time; *h*: total artery scan height; *n*: number of height steps.

On the other hand, paraffin embedding resulted in cracks within the plaque and air inclusions within the soft tissue, where phase wrapping appears as a result of the phase shift difference between materials exceeding 2*π*, leading to streak-like artefacts, see Fig. 1b. These artifacts were previously detected, see for example the images of Fig. 5 in^8^. At the selected photon energy, streak artefacts due to high absorption of the hard tissue components are not observed in the absorption-contrast data. A direct comparison of the diseased artery before and after paraffin embedding clearly indicates that only the plaque is preserved in size and shape, and the soft tissues substantially deform in a non-isotropic manner.

The image in Fig. 1c displays a slice selected from the CT Dataset #3*A* obtained from the decalcified artery. Decalcified non-dehydrated artery segments were not considered, because the case is not part of the workflow in histology. The decalcification process substantially reduces the plaque, in which case streak artefacts are prevented. However, additional morphology modifications are present, including damage to the inner and outer parts of the vessel wall (data not shown). Lumen segmentation failed where damage of the vessel wall was present. For comparison, the related slice from absorption contrast data, i.e. Dataset #3*B* (see Fig. 1d) with around two times worse spatial resolution is displayed. Likewise, this image indicates that the lumen cannot be segmented by means of the presently available automatic procedures.

The gradients in intensity and the large deviation from the cylindrical shape prevent the successful lumen segmentation by means of Frangi filtering^32^, which is widely employed as vessel detector in 3D imaging. In order to determine the cross-section along the artery, the lumen has been identified and thus segmented in undamaged parts, involving user interactions (*cf*. Methods, Lumen segmentation).

### Laboratory-based μCT vs. synchrotron-radiation-based μCT imaging

**Figure 2:**
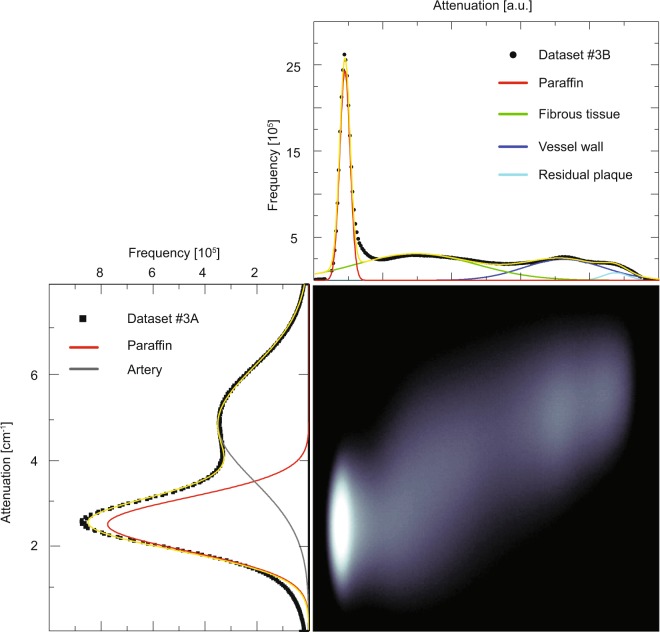
Joint histogram and individual histograms of the registered volumes from Datasets #3*A* (bottom, left) and #3*B* (top, right). The peaks of the histograms were fitted with multi-Gaussian distributions (yellow curve). Whereas the individual histograms are given by a linear scale, the joint histogram is plotted on a logarithmic scale. Adapted from the manuscript of M.B.’s thesis^46^.

**Figure 3:**
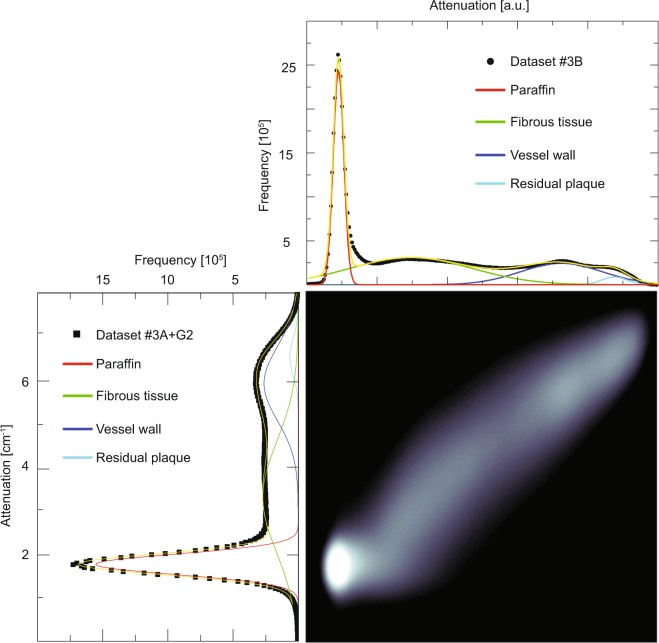
Joint histogram and individual histograms of the registered volumes from Datasets #3*A* after applying a Gaussian filter (bottom, left) and #3*B* (top, right). Filtering Dataset #3*A* gave rise to sharper peaks, which overlapped without filtering (see Fig. 2, bottom left). Adapted from the manuscript of M.B.’s thesis^46^.

A bivariate representation of the histograms from the registered Datasets #3*A* (bottom, left) and #3*B* (top, right) is displayed in Fig. 2. This joint histogram contains four clusters, each corresponding to the embedding material (paraffin), the fibrous tissue, the vessel wall, and the residual plaque, respectively. In both histograms, the paraffin (red-colored Gaussian peak) is clearly present and segmentable. It is, however, broader in the histogram of the Dataset #3*A* than in the histogram of Dataset #3*B*. The histogram of Dataset #3*B* distinguishes, in addition to the paraffin, between fibrous tissue (green-colored Gaussian peak), tissues forming the vessel wall (dark blue-colored Gaussian peak), and the remaining calcification (light blue-colored Gaussian peak), whereas the histogram of Dataset #3*A* displays a broad peak (gray-colored Gaussian peak). After filtering Dataset #3*A* using a Gaussian with *σ* = 2 pixels, the peaks in the histogram also exhibit the four Gaussian peaks, as exemplified in Fig. 3 bottom left.

### Complementarity of tomographic imaging to histology

**Figure 4:**
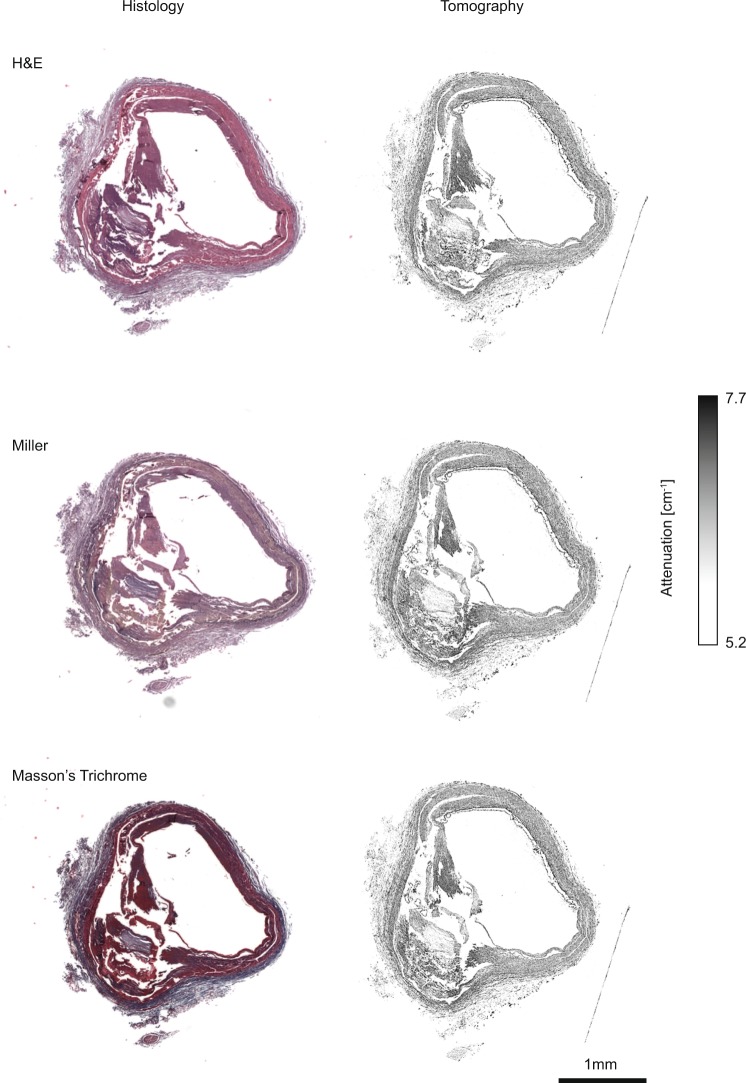
Comparison of histology slices and their CT counterparts from Dataset #3*A*. The selected micrographs of the histological sections (left) of the decalcified human coronary artery were stained using H&E, Miller, and Masson’s Trichrome. Tomography slices (right) matching the histological sections were identified by automatic registration. Adapted from the manuscript of M.B.’s thesis^46^.

**Figure 5:**
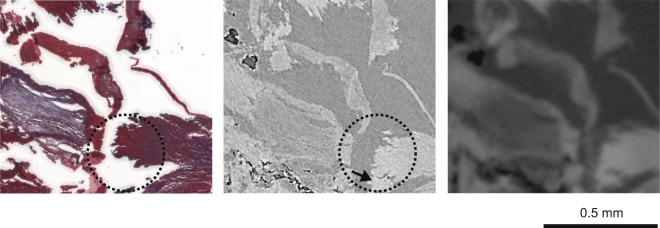
Magnified parts of the slices shown in Fig. 4. The histological slice, stained by Masson’s Trichrome (left), exhibits anisotropic deformation resulting from the cutting process, as recognizable by comparison with the counterparts from the Datasets #3*A* (middle) and #3*B* (right). After histological sectioning, part of the tissue drastically deforms (dashed black circle) or is lost (black arrow). Adapted from the manuscript of M.B.’s thesis^46^.

Optical micrographs of three histological slices, which were stained using H&E, Miller, and Masson’s Trichrome are displayed on the left of Fig. 4. The corresponding CT-slices from the Dataset #3*A* (Fig. 4, right) were selected on the basis of slice-to-volume registration^33^. On the images of the histological sections, one finds the adventitial tissue on the outer part of the vessel wall. Muscular media is stained red-brown on H&E, gray on Miller and red-blue on Masson’s Trichrome. Asymmetric decalcified plaque is visible on the left side of the artery. The artefacts caused by histological slicing are clearly visible, if one compares them with the tomography data (*cf*. also clipped slices from Fig. 4 displayed in Fig. 5). The artery walls, and especially the region where the decalcified plaque is present, are deformed.

### Centerline extraction and artery lumen cross-section determination

To extract the centerline perpendicular to the cross-sections of successfully segmented lumen, a dedicated iterative procedure,for handling large deviations from the cylindrical shape was implemented (see section Methods).

**Figure 6:**
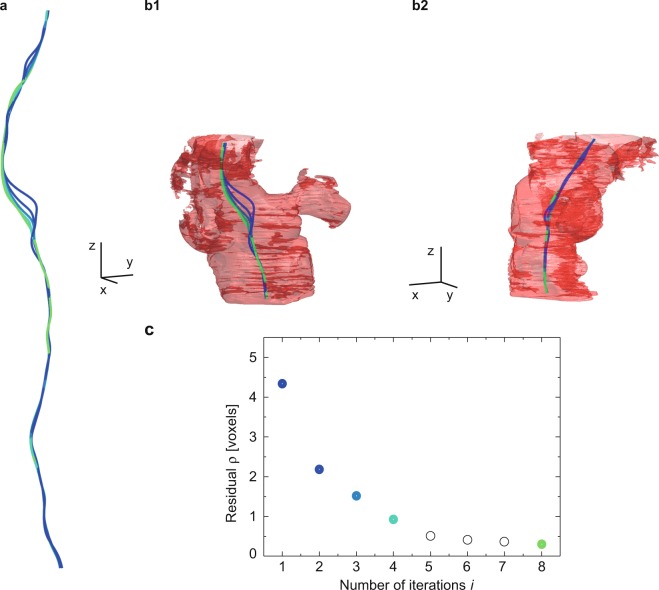
Rendering of the computed centerlines and the related residual *ρ*. Results for the centerlines obtained from the first to the fourth as well as the eighth iterations, derived from Dataset #1, are shown in colors ranging from dark blue to light green (**a**). Part of the centerlines from (**a**) and the corresponding lumen are viewed from two perspectives (**b1**,**b2**). The color-coded residuals are plotted vs. the iteration steps (**c**). Iterations 5 to 7 are not represented in (**a**), and the orthogonal axes of the scale bar correspond to a length of 1 mm each. Adapted from the manuscript of M.B.’s thesis^46^.

The centerline found after the first four (*i* = 1, 2, 3, 4) and the eighth (*i* = 8) iterations for the segmented lumen of Dataset #1 are presented in Fig. 6a.

Figure 6b1 displays the centerline plotted within the corresponding artery lumen, oriented as in Fig. 6a, and rotated by 90° as shown in Fig. 6b2. The appendage shown in Fig. 6b1, represents a bifurcation present along the artery. Due to the artery bifurcation, the centerline *i* = 1, a dark blue-colored curve, exhibits a bulge that disappears at iteration *i* = 8, a light green-colored curve, where the condition *ρ* < *μ* is reached (*μ* = 0.3 voxel). Convergence of the residual *ρ* for Dataset #1 is represented in Fig. 6c.

**Figure 7:**
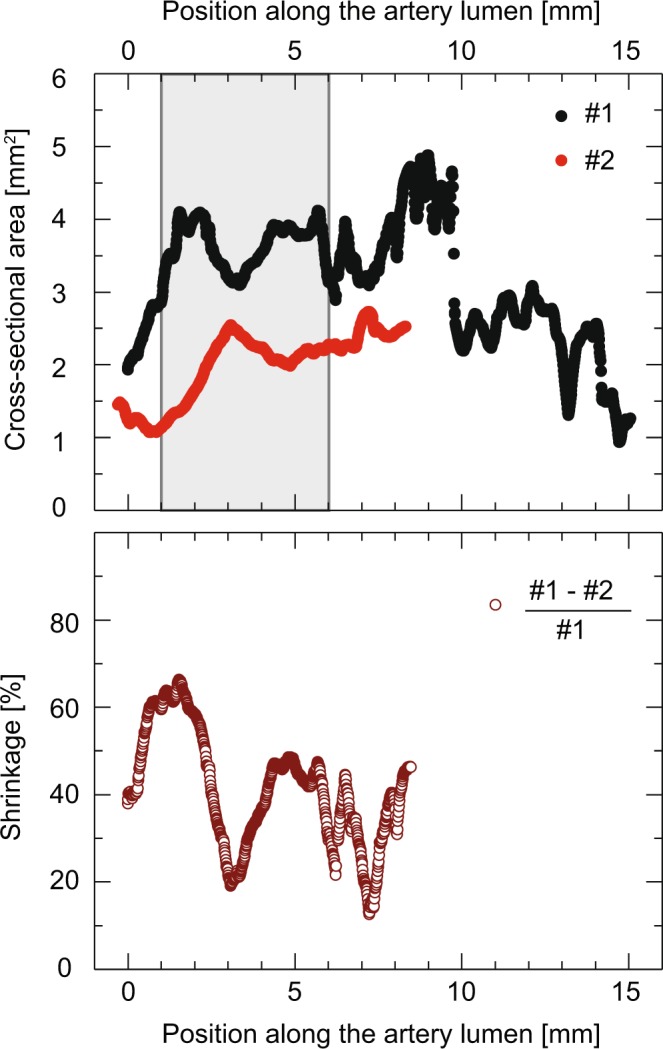
Cross-sectional area obtained from Datasets #1 (black dots) and #2 (red dots) along the segmented artery lumen, and related shrinkage (brown hollow dots). The reduction of the cross-sectional area in Dataset #1 allowed for localizing the plaque-related constriction (top panel). Non-uniform shrinkage (bottom panel) as a result of paraffin embedding prevented any meaningful determination of stenosis. Using the plaque, the two datasets were perfectly aligned. The gray-shaded box (top panel) indicates the location of the data represented in Fig. 8. Adapted from the manuscript of M.B.’s thesis^46^.

**Figure 8:**
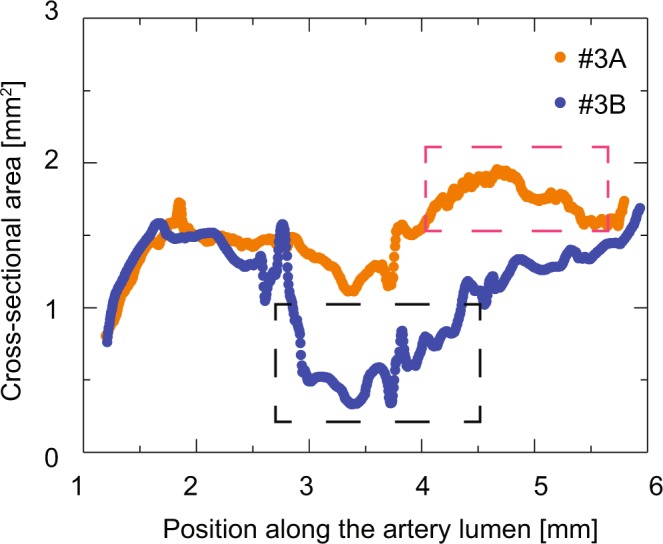
Cross-sectional area along the artery as derived from Dataset #3*A* (orange dots) and Dataset #3*B* (blue dots). The cross-sectional areas overlap from position 1.2 to 2.6 mm. Damage introduced during the re-embedding procedure hampered the lumen segmentation of both datasets. Red- and black-dashed boxes indicate regions where the lumen cross-section was either overestimated (Dataset #3*A*) or affected by 20-voxel-wide erosion/dilation (Dataset #3*B*). Adapted from the manuscript of M.B.’s thesis^46^.

Figure 7 shows the cross-sectional areas of Datasets #1 (black dots) and #2 (red dots) obtained as described in Methods section. The procedure was successfully applied to Dataset #1 for a length of approximately 15 mm out of 20 mm. The remaining artery of 5 mm length suffered from strong intensity gradients. Furthermore, the intensity gradient (see Fig. 1a) strongly affected lumen segmentation in the region between 8 and 10 mm. In Dataset #2, the artery could be segmented with success at a length of 9 mm that remained undamaged after paraffin embedding. The lower diagram in Fig. 7 shows the shrinkage of the artery as a function of the position along the centerline, whereby paraffin embedding caused a reduction in the cross-sectional area by values between about 15% and 65%. The diagram in Fig. 8 shows the results of the cross-sectional areas of Datasets #3*A* (orange dots) and #3*B* (blue dots). The two Datasets #3*A* and #3*B* are acquired from the same specimen measured using synchrotron radiation and conventional source in absorption contrast, respectively.

The comparison demonstrates that the data provide reliable values in a restricted part of the sample, namely from 1.2 to 2.6 mm. From position 4.5 to 5.6 mm (red-colored dashed line), the cross-section obtained from Dataset #3*A* was affected by artefacts, which give rise to an overestimation of the cross-sectional area. From position 2.6 to 4.5 mm (black dashed line), the cross-section of Dataset #3*B* was affected by 20-voxel-wide erosion/dilation, as a consequence of the results provided by lumen segmentation.

## Discussion

For the simultaneous imaging of soft and hard tissue components the phase-contrast grating-based approach^34^ has been selected, because even the use of suitable staining protocols cannot eliminate the strong dependence of X-ray absorption from the atomic number of the constituents.

Nevertheless, the high phase shift of the plaque still gives rise to sporadic streak-like artefacts as clearly present in Datasets #1 and #2 (*cf*. Fig. 1a,b).

It has been reported that formalin fixation causes tissue shrinkage of about 3–6%^35^. In the brain, formalin fixation gives rise to local strains as large as 15%^36^. Therefore, one can reasonably assume that the derived lumen of the artery segment will be smaller than in the *in vivo* situation. Highly intense hard X rays often induce the formation and growth of air bubbles, especially at internal interfaces^23^, which are found within the vessel walls (*cf*. Dataset #1), causing severe changes in the Δ*δ* values.

Air bubble formation is circumvented by paraffin embedding, albeit this does lead to streak-like artefacts caused by cracks in the plaque and entrapped air in the soft tissue during the embedding process. H. J. Gundersen *et al*. have reported that dehydration, owing to paraffin embedding, can result in shrinkage of up to 30%^37^. In fact, the data represented in Fig. 1b confirms that paraffin embedding causes drastic deformation and shrinkage of the soft tissues while the morphology of the plaque remains unaffected.

The streak artefacts herein vanished as the result of the decalcification (*cf*. Fig. 1b), although lumen segmentation was hampered, as remains of the decalcified plaque infiltrated the lumen (data not shown). The morphology of the diseased part of the artery could therefore not be reliably quantified.

In order to investigate the impact of the individual tissue preparation steps, namely formalin-fixation, paraffin embedding and decalcification, the cross-sectional area along the artery lumen has to be determined. Since the artery cannot be reasonably described as a cylinder, its lumen was not parallel to the *z*-axis. Determining the center of each CT-slice is insufficient for extracting the centerline. Approaches reported in the literature, including piece-wise linear curves^38^ and B-splines^39,40^, are successful only to a limited extent due to the challenging geometry. A medial axis transformation algorithm^26^, often employed to extract the centerline from non-cylindrical objects, was applied without success.

Hence, it was necessary to search for alternative ways to extract the centerline along the bent and bifurcated diseased artery. The proposed algorithm revealed strong convergence and avoids large jumps compared to piece-wise linear curves or B-splines as shown in a benchmark problem considering a thin geometry with local swellings (see Fig. S1, Supplement). Application of the algorithm to the individual datasets indicates that the number of iterations depends not only on deviation from the cylindrical shape but also on the specimen preparation. For the Datasets #2 and #3*A*, the number of iterations was four and five, to reach a residual of less than 0.3 voxel, respectively, whereas for the Dataset #1 the artery bifurcation increased the number of iterations to eight (see Fig. 6). Dataset #3*B* contained some tissue debris in the segmented lumen, resulting after the decalcification process. This dataset was subjected to 20 voxel-wide erosion/dilation. The number of iterations to reach convergence increased to 29.

If one assumes that tissue shrinkage owing to formalin fixation is below 10%, as reported by Zehbe *et al*.^35^, the plot of the cross-section along the centerline for Dataset #1 (Fig. 7, black dots) should yield the information on the artery lumen which reduces due to the formation of the plaque within the vessel wall. Contrary to formalin fixation, embedding the specimen in paraffin required substantial handling by the user, leading to deformation which influenced the morphology of the specimen, and thus the lumen cross-section (*cf*. Fig. 7, red dots). In particular, the artery segment was embedded, de-embedded and re-embedded several times before obtaining a paraffin block without air bubbles entrapped.

The histogram data for the synchrotron radiation source-based μCT does not allow distinguishing the information on the fibrous tissue, the vessel walls and the remaining plaque, which is instead possible observing the histogram data from the conventional X-ray source. As the filtering of the data acquired at the synchrotron radiation source highlights the peaks, the authors suspect that the proprietary Bruker reconstruction software contains a similar filtering feature.

Each modality gives rise to four components, namely the vessel wall, the fibrous tissue, the remaining plaque, and the embedding material (paraffin).

To validate the tomography data, histology is required. In the current literature, it has been claimed that with respect to H&E and Miller, Masson’s Trichrome staining is the better choice for comparing histology with tomography data^8^. In the present study, no significant differences in terms of tissues identification could be found, so the arbitrary selection of one staining protocol was enough to validate the morphological findings from hard X-ray tomography. Although the features are co-resident in tomography and histology, one recognizes some additional deformations owing to cutting.

The precise determination of the lumen from a plaque-containing human artery remains a subject for further research, since (i) the *in-vivo* methods do not reach the necessary micrometer resolution, (ii) the *post*-*mortem* evaluation using hard X rays is not only amendable because formalin fixation results in moderate shrinkage, but also because it often induces the formation of bubbles, which can grow in size and, therefore, locally deform the tissue during the data recording, (iii) when embedding the diseased artery into paraffin, bubble formation and growth can be prevented at the expense of massive local deformations, (iv) decalcification prevents the occurrence of streak artefacts, but often also induces tissue damage, which seriously compromises lumen extraction, and (v) the histological sections of decalcified arteries hardly represent the artery’s morphology *in vivo*.

In conclusion, the present study proposes that the lumen from a plaque-containing artery should be based on formalin fixation and hard X-ray imaging. The formation and growth of gas bubbles in the formalin-fixed specimen can be reduced by using less intense X-ray beams with a photon energy as high as possible and adapted protocols for preparing the formalin solution and the tissue. In addition to the already mentioned strategies to overcome air bubble formation and growth, it is beneficial to perform phase-contrast tomography using a bench-top system as done by Vågberg *et al*.^22^ A limitation of the present study is that the analysis pipeline was only demonstrated for one selected atherosclerotic artery segment and on a benchmark problem for centerline extraction. Future research activities may include besides a reduction of imaging and preparations artefacts, a confirmation on a substantially larger number of specimens and a generalization of the proposed processing pipeline for further biomedical applications.

## Methods

### Specimen preparation

A 2.2-cm-long segment of a plaque-containing human coronary artery from the distal part of the anterior interventricular artery was explanted *post*-*mortem* from a female patient. *Ante*-*mortem* she consented to give her body for research purposes to the medical faculty of Lausanne University, Switzerland, the forensic department of which is a joint venture with the neighboring University of Geneva. Ethical approval for this study (Ethical Committee N° NAC 09-105) was provided by the Ethical Committee N.A.C. (Neuclid, Apsic, Chirurgie, Pathologie, Radiologie) of Geneva University Hospitals, Geneva. Informed consent for scientific use was obtained from all participants and all methods were performed in accordance with relevant guidelines and regulations.

The artery segment was placed in a 2.0 mL Eppendorf tube and fixed with 4% paraformaldehyde (PFA). After imaging at the synchrotron radiation facility, the segment was embedded in paraffin. Prior to paraffin embedding, the surrounding tissues, mainly fatty tissue, were removed from the artery. To minimize the specimen diameter, the paraffin block was trimmed with a scalpel.

Later, the segment was de-embedded and then subjected to decalcification. The segment, fixed in 4% paraformaldehyde (PFA) for two days, was immersed in a decalcifying solution (87 vol% distilled water, 8 vol% formic acid, 5 vol% PFA) at a temperature of 37 °C. Decalcification was stopped when the decalcifying solution did not become white after mixing with 1 mL ammoniumoxalate (5%, vol/vol) and 1 mL ammonia (5%, vol/vol). Subsequently, the specimen was immersed in alcohol 70% for a period of five hours, dehydrated, embedded in paraffin at a temperature of 60 °C, and cooled down to room temperature.

### Multimodal imaging of a plaque-containing human coronary artery

For simultaneous visualization of the highly X-ray absorbing plaque and the surrounding soft tissues, the human coronary artery segment was imaged using XGI-based μCT at the beamline P07 (PETRA III, DESY, Hamburg, Germany) once without and once with paraffin embedding. After decalcification and re-embedding into paraffin, the segment was visualized using the absorption-contrast-based tomography setup at the beamline P05 (PETRA III, DESY, Hamburg, Germany) and using the laboratory-based tomography system Skyscan 1275 (Bruker, Kontich, Belgium). Table 1 summarizes the preparation of the human artery segment and the parameters employed for data acquisition.

#### Laboratory-based tomography

Radiographs were recorded using a 3 Megapixel (1944 × 1536) CMOS camera featuring a pixel size of 75 μm. In total, 1200 projections (rotation angle increment of 0.3°) of the specimen were collected using an acceleration voltage of 15 kV and a beam current of 156 μA. Exposure time was set to 2.3 s. Effective pixel length corresponded to 5.2 μm. The tomograms were reconstructed using the manufacturer’s software NRrecon, in which the Feldkamp algorithm^41^ is implemented.

#### Synchrotron radiation-based double-grating interferometry

The P07 beamline is operated by Helmholtz-Zentrum Geesthacht, Germany. For phase imaging, the specimen was placed in a water bath to reduce artefacts owing to large X-ray wave front curvature at the specimen background interface, commonly referred to as “phase-wrapping” artefacts. As the size of the artery exceeded the field of view, it was moved vertically to the X-ray beam in 10 and 13 height steps for the formalin-fixated and paraffin-embedded states, respectively. Radiographic data were acquired at a photon energy of 45 keV. The double-crystal Si(111) monochromator (horizontal Laue geometry) was bent to match Rowland circle geometry. The beam-splitter grating (4.8 μm periodicity, Ni) was placed 31.4 cm away from the analyzer grating (2.4 μm periodicity, Au), corresponding to the third fractional Talbot order. The detection unit contained a 100 μm-thick CdWO_4_ scintillator. The obtained optical image was magnified 5× and recorded by a CMOS camera, which was developed and produced at the Institute for Data Processing and Electronics (Institute of Technology, Karlsruhe, Germany) and uses a chip (CMOSIS, Antwerp, Belgium) with 20 megapixels (5120 × 3840) each 6.4 μm in size. The effective pixel length corresponded to 1.3 μm. For the formalin-fixed specimen, 900 projections were recorded over 360°, using an asymmetric rotation axis configuration, whereas 1200 projections were acquired for the paraffin-embedded specimen. At each angle, four phase-step images were taken over one period of the interference pattern. The exposure time was set to 0.2 s per phase step image. Prior to reconstruction, the phase tomograms were binned by factors of 2, 4, and 8 to simplify their handling and to improve the contrast^42^. After binning, phase-retrieval was performed by means of pixel-wise Fourier analysis. In order to reconstruct the phase tomograms, the differential data were integrated and then treated like attenuation-based data.

#### Synchrotron radiation-based tomography in absorption-contrast mode

The undulator source combined with the double-crystal monochromator, consisting of two Si(111) Bragg crystals, provided photons with an energy of 10 keV at the HZG beamline P05. The X-ray photons were converted into an optical image by a 100 μm-thick CdWO_4_ scintillator and recorded by a camera (SciCam series) with a Kodak CCD chip KAF-09000 (3056 × 3056) comprising of 12 μm-wide pixels. The specimen-detector distance was set to 10 mm. In all, 1200 equiangular radiographs with an effective pixel length of 2.4 μm were recorded along 180°, with an exposure time of 1.5 s per projection. Prior to reconstruction, the projections were binned by a factor of two. The tomograms were obtained by the standard filtered back-projection algorithm.

### Lumen segmentation

The lumen of the artery was determined from the tomography data, by means of the region-growing tool available in VG Studio MAX 2.1 (Volume Graphics GmbH, Heidelberg, Germany). Prior to this segmentation procedure, the data were smoothed using a median filter with a kernel size of 15. In order to evaluate the impact of the median filter to the segmented lumen, the difference of the tomography data before and after the application of the median filter has been determined. The results showed an enlargement of the lumen cross-section by one pixel (see Fig. S2, Supplement). In case of Dataset #3*B* the segmented lumen was eroded/dilated by 20 voxels.

### Cross-section along the centerline determined from the segmented lumen

**Figure 9:**
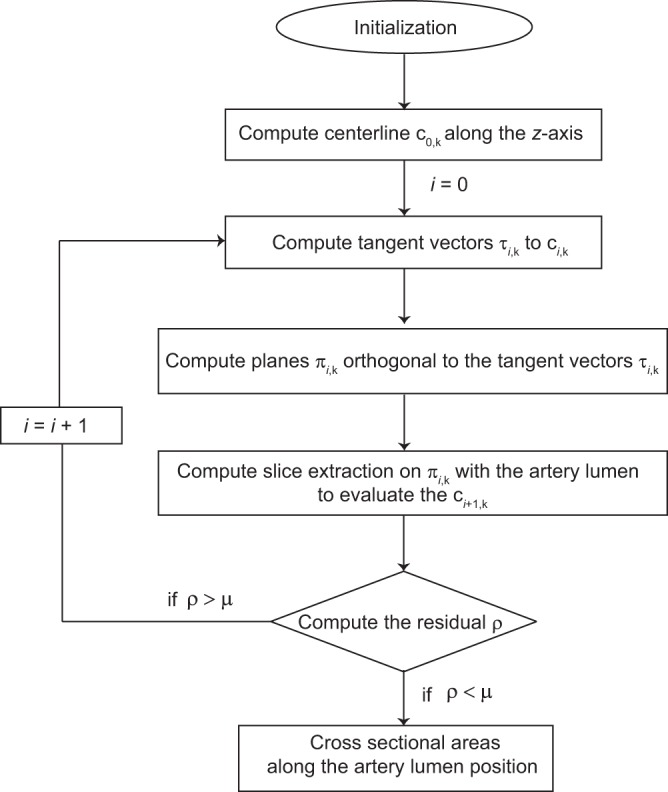
Flow chart describing the implemented iterative procedure. The flow chart shows the main steps employed to determine the centerline and the cross-sectional area along the artery lumen. Adapted from the manuscript of M.B.’s thesis^46^.

For the datasets listed in Table 1, the centerline of the artery segment was calculated. The individual steps in the iterative procedure used herein are represented in Fig. 9. The main steps are as follows:

1. **Initialization**. In the initialization step, the binarized data for the segmented artery lumen were loaded as a stack of 2D slices in the *x*–*y*-plane and stored as a 3D volume with isotropic resolution in the *x*-, *y*-, and *z* directions. The *z*-axis was oriented parallel to the artery.
2. **Compute centerline** ***c***_0,*k*_ **along the** ***z***-**axis**. The centerline is discretized on points *c*_0,*k*_ with *k* = 1, …, *N*_*k*_, where *N*_*k*_ is the number of *x*–*y*-slices. The points *c*_0,*k*_ correspond to the geometrical centers of the lumen for each slice orthogonal to the *z*-axis, and the resulting centerline *c*_0,*k*_ is smoothed using a Gaussian filter (*σ* = 20 pixels). If the artery lumen is parallel to the *z*-axis, the centerline will be identified and no further steps will be necessary.
3. **Compute tangent vectors** ***τ***_***i***,***k***_ **to** ***c***_***i***,***k***_. Tangent vectors *τ*_*i*,*k*_ to the centerline *c*_*i*,*k*_ are computed using a finite difference second-order method.
4. **Compute planes** ***π***_***i***,***k***_ **orthogonal to the tangent vectors** ***τ***_***i***,***k***_. In this step, the planes *π*_*i*,*k*_ orthogonal to the tangent vectors *τ*_*i*,*k*_ are determined.
5. **Compute slice extraction on** *τ*_*i*,*k*_ **with the artery lumen to evaluate the** ***c***_***i*+1**,***k***_. The slices are extracted using a linear interpolation. Here, a relaxation method is introduced to stabilize convergence for centerline identification. The extracted slices allow for calculating the centerline points *c*_*j*,*k*_. In the next iteration, the centerline *c*_*i*+1,*k*_ is determined by ${c}_{i+1,k}=(1-\beta ){c}_{j,k}+\beta {c}_{i,k}$, setting the relaxation parameter beta to $\beta =0.5$ for the Datasets #1, #2, and #3*A* and $\beta =0.9$ for the Dataset #3*B* and finally smoothed using the Gaussian filter. Thus, the residual *ρ* of the centerline *c*_*i*−1,*k*_ and *c*_*i*,*k*_ is computed, being $\rho ={\sum }_{k=1}^{{N}_{k}}\,d({c}_{i-1,k},{c}_{i,k})/{N}_{k}$ and *i* the number of iterations. If *ρ* > *μ*, the algorithm returns to step 3, with the threshold *μ* = 0.3 voxel length being well below one voxel length; otherwise, the iteration loop is terminated and the centerline *c*_*final*,*k*_, the tangent vectors *τ*_*final*,*k*_, and the planes *π*_*final*,*k*_ are found.
6. **Cross**-**sectional areas along the artery lumen position**. In this step, slices orthogonal to the tangent vectors *τ*_*final*,*k*_ are extracted from the volumetric data of the artery lumen and their cross-sectional areas computed.

### Histology

Histological sections were prepared from the decalcified artery according to a standard protocol^8^. Briefly, three slices, each about 2 μm thin, were cut along the artery. The slices were colored with three selected stains, namely hematoxylin and eosin (H&E), Miller or Masson’s Trichrome, and mounted on glass slides. Images of the histological slides were taken using the Panoramic MIDI scanner (3DHistech, Sysmex Suisse) with a pixel resolution of 0.24 μm.

### Data registration

One height step selected from Dataset #1 was successfully registered to the corresponding height step of Dataset #2, using an affine registration algorithm^43^. Three-dimensional registration was carried out using the classical maximization of mutual information^44,45^ (see CT-slices in Fig. 1a,b). The same procedure was applied to the Datasets #3*A* and #3*B* (see CT-slices in Fig. 1c and 1). To match the histology and tomography data (#3*A*), slice-to-volume registration^33^ was performed. To facilitate the comparison with the tomography data, and prior to registration, the histology images were binned by a factor of 20 and converted to grayscale.

## Supplementary information

Centerline extraction on a benchmark problem and effect of the median filter on the lumen area.

## References

[1] Doriot P-A (2000). *In*-*vivo* measurements of wall shear stress in human coronary arteries. Coron. Artery Dis..

[2] Cheng C (2007). Large variations in absolute wall shear stress levels within one species and between species. Atherosclerosis.

[3] Keaney JF (2000). Atherosclerosis: from lesion formation to plaque activation and endothelial dysfunction. Molecular Aspects of Med..

[4] World Health Organisation (WHO). Cardiovascular diseases (CVDs)- Key facts, May 2017, http://www.who.int/cardiovascular_diseases/en/ (last access Feb 12, 2019).

[5] Saxer T, Zumbuehl A, Müller B (2013). The use of shear stress for targeted drug delivery. Cardiovascular Research.

[6] Holme MN (2012). Shear-stress sensitive lenticular vesicles for targeted drug delivery. Nature Nanotechnology.

[7] Hibi K, Kimura K, Umemura S (2014). Clinical utility and significance of intravascular ultrasound and optical coherence tomography in guiding percutaneous coronary interventions. Circulation Journal.

[8] Holme MN (2014). Complementary x-ray tomography techniques for histology-validated 3d imaging of soft and hard tissues using plaque-containing blood vessels as examples. Nature Protocols.

[9] Wintermark M (2008). High-resolution ct imaging of carotid artery atherosclerotic plaques. American Journal of Neuroradiology.

[10] Stalder AK (2014). Combined use of micro computed tomography and histology to evaluate the regenerative capacity of bone grafting materials. International Journal of Materials Research.

[11] Albers J, Markus MA, Alves F, Dullin C (2018). X-ray based virtual histology allows guided sectioning of heavy ion stained murine lungs for histological analysis. Sci. Rep..

[12] Hetterich H (2015). X-ray phase-contrast computed tomography of human coronary arteries. Investigative Radiology.

[13] Hetterich H (2014). Phase-contrast ct: qualitative and quantitative evaluation of atherosclerotic carotid artery plaque. Radiology.

[14] Walton LA (2015). Morphological characterisation of unstained and intact tissue micro-architecture by X-ray computed micro-and nano-tomography. Sci. Rep..

[15] Langheinrich AC (2004). Atherosclerotic lesions at micro ct: feasibility for analysis of coronary artery wall in autopsy specimens. Radiology.

[16] Momose A (2005). Recent advances in x-ray phase imaging. Japanese Journal of Applied Physics.

[17] Momose A (2003). Phase-sensitive imaging and phase tomography using X-ray interferometers. Optics Express.

[18] Bonse U, Hart M (1965). An x-ray interferometer. Applied Physics Letters.

[19] Snigirev A, Snigireva I, Kohn V, Kuznetsov S, Schelokov I (1995). On the possibilities of x-ray phase contrast microimaging by coherent high-energy synchrotron radiation. Review of Scientific Instrum..

[20] Davis T, Gao D, Gureyev T, Stevenson A, Wilkins S (1995). Phase-contrast imaging of weakly absorbing materials using hard x-rays. Nature.

[21] Pfeiffer F, Weitkamp T, Bunk O, David C (2006). Phase retrieval and differential phase-contrast imaging with low-brilliance x-ray sources. Nature Physics.

[22] Vågberg W, Persson J, Szekely L, Hertz HM (2018). Cellular-resolution 3d virtual histology of human coronary arteries using x-ray phase tomography. Sci. Rep..

[23] Lang S (2014). Experimental comparison of grating- and propagation-based hard x-ray phase tomography of soft tissue. Journal of Applied Physics.

[24] Sofka M, Stewart CV (2006). Retinal vessel centerline extraction using multiscale matched filters, confidence and edge measures. IEEE Trans. Medical Imaging.

[25] Behrooz A, Kask P, Meganck J, Kempner J (2017). Automated quantitative bone analysis in *in vivo* x-ray micro-computed tomography. IEEE Trans. Medical Imaging.

[26] Saha PK, Borgefors G, di Baja GS (2016). A survey on skeletonization algorithms and their applications. Pattern Recogn. Letters.

[27] Younas, S. & Figley, C. R. Development, implementation and validation of an automatic centerline extraction algorithm for complex 3D objects. *Journal of Medical and Biological Engineering* 1–21, 10.1007/s40846-018-0402-1 (2018).

[28] Sironi A, Türetken E, Lepetit V, Fua P (2016). Multiscale centerline detection. IEEE Trans. Pattern Anal. Mach. Intell..

[29] Herzen J (2014). Imaging liver lesions using grating-based phase-contrast computed tomography with bi-lateral filter post-processing. PLoS One.

[30] Hieber SE (2016). Tomographic brain imaging with nucleolar detail and automatic cell counting. Scientific Rep..

[31] Müller B (2012). Three-dimensional registration of tomography data for quantification in biomaterials science. Int. J. Mater. Res..

[32] Frangi, A. F., Niessen, W. J., Vincken, K. L. & Viergever, M. A. Multiscale vessel enhancement filtering. *Medical Image Computing and Computer*-*Assisted Intervention* - *MICCAI98* 130–137 (1998).

[33] Chicherova, N. *et al*. Automatic deformable registration of histological slides to *μ*ct volume data. *Journal of Microscopy* 49–61 (2018). 10.1111/jmi.12692 29533457

[34] Momose A, Takeda T, Yoneyama A, Koyama I, Itai Y (2002). Phase-contrast x-ray imaging using an x-ray interferometer for biological imaging. Analytical Sciences.

[35] Zehbe R (2010). Going beyond histology. synchrotron micro-computed tomography as a methodology for biological tissue characterization: from tissue morphology to individual cells. Journal of the Royal Society Interface.

[36] Schulz G (2011). Three-dimensional strain fields in human brain resulting from formalin fixation. Journal of Neuroscience Methods.

[37] Gundersen, H. J. G., Mirabile, R., Brown, D. & Boyce, R. W. *Chapter 8*-*Stereological Principles and Sampling Procedures for Toxicologic Pathologists* 215–286 (2013).

[38] Lacoste, C., Finet, G. & Magnin, I. E. Coronary tree extraction from x-ray angiograms using marked point processes. *Third IEEE International Symposium Biomedical Imaging: Nano to Macro* 157–160 (2006).

[39] Bouix S, Siddiqi K, Tannenbaum A (2005). Flux driven automatic centerline extraction. Medical Image Anal..

[40] Frangi, A. F., Niessen, W. J., Nederkoorn, P. J., Elgersma, O. E. H. & Viergever, M. A. Three-dimensional model-based stenosis quantification of the carotid arteries from contrast-enhanced mr angiography. *Proceedings IEEE Workshop Mathematical Meth*. *in Biomedical Image Anal*. 110–118 (2000).

[41] Feldkamp LA, Davis LC, Kress JW (1984). Practical cone-beam algorithm. Journal of the Optical Society of America A.

[42] Thurner P, Beckmann F, Müller B (2004). An optimization procedure for spatial and density resolution in hard x-ray micro-computed tomography. Nucl. Instrum. Methods Phys. Res. B.

[43] Fierz FC (2008). The morphology of anisotropic 3d-printed hydroxyapatite scaffolds. Biomaterials.

[44] Maes F, Collignon A, Vandermeulen D, Marchal G, Suetens P (1997). Multimodality image registration by maximization of mutual information. IEEE Trans. Medical Imaging.

[45] Viola P, Wells WM (1997). Alignment by maximization of mutual information. International Journal of Computer Vision.

[46] Buscema, M. Evaluation of 100-nm-size mechano-responsive liposomes for targeted delivery at constricted arteries. (PhD thesis, University of Basel, document server edoc.unibas.ch, 2018).

