# Supplementary material for: Ex vivo evaluation of an atherosclerotic human coronary artery via histology and high-resolution hard X-ray tomography
Source: Sci Rep. 2019 Oct 4;9:14348. doi: 10.1038/s41598-019-50711-1 (PMC6778097; doi:10.1038/s41598-019-50711-1)
Supplement: Supplementary file 1 — Centerline extraction on a benchmark problem and effect of the median filter on the lumen area. [file 41598_2019_50711_MOESM1_ESM.docx]

**Ex vivo evaluation of an atherosclerotic human coronary artery via histology and high-resolution hard X-ray tomography**

Marzia Buscema^1^, Simone E. Hieber^1^*, Georg Schulz^1^, Hans Deyhle^1^, Alexander Hipp^2^, Felix Beckmann^2^, Johannes A. Lobrinus^3^, Till Saxer^4^, and Bert Müller^1^

**Affiliations**

^1^Biomaterials Science Center, Department of Biomedical Engineering, University of Basel, Allschwil, Switzerland.

^2^ Institute of Materials Research, Helmholtz-Zentrum Geesthacht, Geesthacht, Germany.

^3^ Neuropathology Unit, University Hospital of Geneva, Geneva, Switzerland.

^4^Faculty of Medicine, University of Geneva, Geneva, Switzerland.

***Corresponding author**: simone.hieber@unibas.ch

**Supplementary Figures**

**
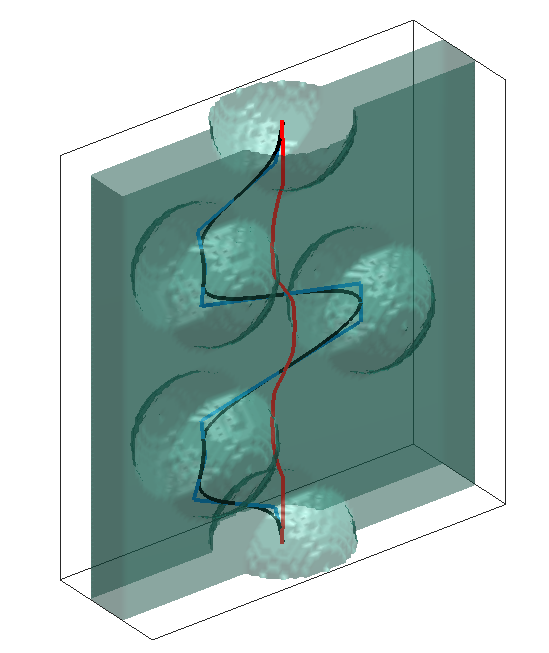
**

**Supplementary Figure S1: Centerline extraction on a benchmark problem.** Centerlines extracted by a piece-wise linear approach (blue) and B-splines (dark green) and the proposed approach (red) on a benchmark problem. The regular thin geometrical shape is challenging due to local swellings leading to large jumps using standard approaches. The proposed approach provides a suitable centerline located in the middle.


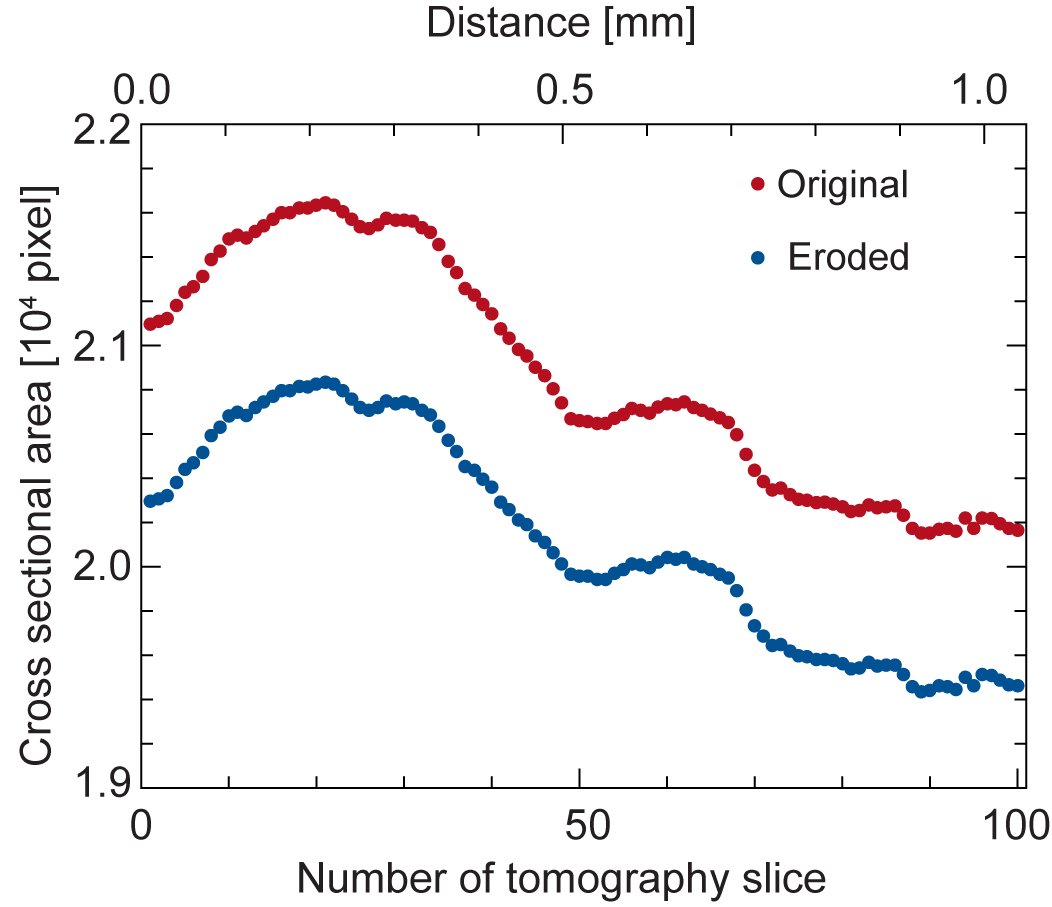


**Supplementary Figure S2: Effect of the median filter on the lumen area.**

Hundred tomography slides from Dataset #2 segmented after applying a median filter with a kernel size of 15 (red dots). Data filtering caused constant lumen enlargement over the selected CT-slides, and therefore the lumen cross-section was eroded by one pixel (blue dots). Adapted from the manuscript of M.B.’s thesis, University of Basel 2018.
